# New Approaches and Technologies in Orthodontics

**DOI:** 10.3390/jcm13092470

**Published:** 2024-04-24

**Authors:** Letizia Perillo, Fabrizia d’Apuzzo, Vincenzo Grassia

**Affiliations:** Multidisciplinary Department of Medical-Surgical and Dental Specialties, University of Campania Luigi Vanvitelli, 80138 Naples, Italy; letizia.perillo@unicampania.it (L.P.); fabrizia.dapuzzo@unicampania.it (F.d.)

## 1. Introduction

In recent years, new diagnostic and treatment approaches in orthodontics have arisen, and there is thus a need for researchers and practitioners to stay up to date with these innovations [1,2,3].

In recent years, artificial intelligence (AI) development has advanced, allowing each expert to provide better alternatives to patients, to make more precise diagnoses, and to reduce treatment costs and timeframes [4,5].

New technologies and procedures, such as intraoral scanners, digital models, and measurements, along with increasing use of CBCT [6], have slowly expanded, enhancing overall patient care, treatment plan management, and prevention, particularly in the early stages [7,8].

The everyday use of AI technology has increased the potential of each treatment step in real time, including examining the outcome, evaluating all feasible options, and staging the state of oral hygiene.

Currently, impacted teeth, facial deformities [9], OSA [10], and other oral disorders or pathologies [11] may be detected more quickly, allowing for more direct and timely communication while also enhancing patient compliance [12]. Furthermore, technology has enabled the accurate timing and positioning of many types of orthodontic devices, such as functional appliances and mini-screws, as well as the ability to determine which factors are connected to the orthodontic treatment movement (OTM) [13,14].

The literature is regularly updated with fresh research, allowing doctors to provide more effective and less onerous therapies to each patient [15]. As a result, the goal of this Special Issue was to encourage research in all areas of orthodontics, with an emphasis on diagnostic and therapeutic advances to widen our knowledge and support scientific and clinical discoveries.

## 2. An Overview of Published Articles

Kanavakis G. et al. [16] evaluated the effects of overjet (OVJ) and overbite (OVB) on the profile of middle-aged patients. They concluded that OVJ significantly leads to changes to upper lips, while OVB has minor consequences for the general shape of the profile. However, considering the variety of genetic and environmental factors that affect the soft tissues of the face, these outcomes could become more significant.

A younger sample size, from 12 to 17 years, was analyzed by Bungău T.C. et al. [17], who examined the rejection rate of mini-implants in orthodontics. The application of these devices can be helpful in several approaches according to the severity of the malocclusion. The authors investigated the rejection rate up to three months after positioning. In conclusion, mini-implants showed the highest percentage of rejection (25%) in the buccal mandibular bone during the first month, while in the second month, it was recorded in the lingual region. Thus, mini-implants are useful devices during orthodontic treatment (OT), but their stability must be improved.

Malocclusions have also been studied by other authors. Frutos-Valle L. and colleagues [18] established a Class III skeletal malocclusion sub-phenotype characterization based on proportional cranial measurements using principal component and cluster analyses. They recognized four phenotypic subgroups, from C1 to C4, according to the severity of the malocclusion. Therefore, the authors provided a new subset of Class III skeletal malocclusions, improving diagnostic and therapeutic approaches to this malocclusion.

Similarly, Class III malocclusion was analyzed by Steegman R.M. et al. [19]. This group presented a prospective controlled study to assess the skeletal effect of anchored bone extension (BAMP) in Class III patients with a cleft using CBCT. Their results, with a follow-up of about 3.5 years, provide the first evidence to support BAMP as an effective and reliable treatment option for growing subjects with mild-to-moderate Class III malocclusion up to an average age of 15 years.

The potential use of CBCT was also considered by Griswold O. et al. [20], who conducted a longitudinal retrospective study to compare patients treated only with rapid maxillary expansion (RME) and patients treated with RME and LB using CBCT. Pre- and post-treatment CBCTs were superimposed to determine changes in anterior lower teeth. Both study groups showed no significant changes in the proclination of the lower incisors. Hence, the use of LB would seem not to affect the position of the mandibular incisors significantly.

CBCT was used by other researchers for their studies as well, including Solano Mendoza P. et al. [21], who assessed skeletal and dental changes after mini-screw-assisted rapid palatal expansion (MARPE) in adolescent patients. They conducted an uncontrolled prospective study on 17-year-old subjects with transverse maxillary deficiency. Pre- (T1) and post-expansion (T2) CBCT and digital casts were taken to evaluate changes to the premolar and first molar areas. Therefore, they concluded that MARPE is a successful means of obtaining skeletal maxillary expansion in adolescents, observing only small dentoalveolar changes that are not clinically detectable.

Similarly, Li C. et al. [22] created an efficient and accurate orthodontist-friendly protocol to segment the mandible while maintaining access to internal structures based on CBCT images. At the end of the measurements, it was seen that the mandibular bones of all tested DICOM files were successfully segmented. In addition, all anatomical structures were found in voxel-based overlap, demonstrating the ability of CBCTs to conduct precise internal structure analysis.

Finally, also Kochhar A.S. et al. [23] conducted a retrospective study to compare the accuracy of the identification of reference points and their reproducibility using 3D cephalograms derived from CBCT and digital lateral–lateral radiographs in patients with cleft lip and cleft palate. The identification of the points and their reproducibility on CBCT were found to be statistically significant compared to the lateral–lateral radiographs.

Several digital tools have been employed by Park S.H. et al. [24]. They evaluated the reliability, reproducibility, and validity of orthodontic measurements, such as tooth width, arch length, and arch length discrepancy, on plaster models (P), digital scanning models (MSD), and intraoral scanned digital models (ISD). Most orthodontic measurements have shown high validity. Measurements based on the digital program appeared highly reliable, reproducible, and accurate compared to conventional measurements. Despite this, clinicians should be aware of the errors induced by the distortion caused by using digital models.

Moreover, Gkantidis N. and colleagues [25] developed a 3D overlay technique to evaluate morphological changes in dental surfaces other than occlusal ones. It has been seen that this new technique offers a convenient, accurate, and risk-free assessment of tooth wear. Similarly, Keilig et al. [26] evaluated the efficiency of teeth alignment with clear aligners (CAs) using the 3D overlap, considering the different variability. They deduced that CAs can implement tooth movements effectively through the inclination of clinical crowns. However, digital planning needs to take individual patient parameters into account to make OT more predictable and efficient.

The use of digital instruments and manual techniques was compared by Gera A. et al. [27], who evaluated the validity and reproducibility of the peer assessment rating (PAR) index and its components using software versus a manual method. There were no significant differences in average PAR scores between both methods, but statistical tests confirmed the excellent validity and reproducibility of the PAR index on digital models compared to manual scoring on equivalent printed models through a gauge digital model.

Another field of research was addressed by Templier L. and coworkers [28], who conducted literature research to evaluate and summarize current scientific data on the effectiveness of both adenotonsillectomy (AT) and OT with rapid maxillary expansion (RME) and/or mandibular advancement (MA) in children with obstructive sleep apnea (OSA). They concluded that AT, together with RME and/or MA, is an efficient treatment in pediatric patients with OSA. AT, RME, and/or MA, when performed together, lead to a decrease in the hypopnea apnea index (AHI) and respiratory disorders index (RDI) and an increase in oxygen saturation and the oxygen desaturation index (ODI). However, the risk of relapse may occur even after proper treatment, so myofunctional therapy (MT) should also be implemented during follow-up.

The effects of functional appliances (FA) on upper airway dimensions were analyzed by Bidjan D. et al. [29] through a systematic review and meta-analysis. They gathered 20 non-randomized clinical trials, according to which orthopedic treatment with FA, both fixed and removable, is associated with an increased volume of the oropharynx and nasopharynx compared to natural growth. Removable FAs showed noticeably greater effects compared to fixed ones. In addition, the patient’s age and treatment duration also significantly affected the outcome of FAs on the respiratory tract. Although nasal obstruction (NO) during growth causes the suppression of maxillofacial growth, it is unclear whether the elimination of NO differentially affects maxillary and mandibular growth.

The NO was also investigated by Keitoku M. and colleagues [30]. They performed a study on male mice, removing the sutures for resume nasal breathing to assess if elimination of the NO could allow for normal maxillofacial growth, determining, at the same time, the right timing of intervention. They have, therefore, evaluated immunohistochemical changes in the hypoxia-inducible factor (HIF)-1α, osteoprotegerin (OPG), and nuclear factor receptor activator kappa-B ligand (RANKL) of condylar cartilage. Their study suggests that the elimination of NO is effective in recovering maxillofacial growth. In addition, the optimal timing of surgery differed between the jaw and the jaw.

Other studies have focused on biomarkers. In particular, Luchian I. and collaborators [31] have evaluated the effects of periodontal treatment (PT) alone or in combination with OT on the levels of MMP-9. The results showed that both PT and OT significantly improved clinical parameters and lowered MMP-9 levels compared to the control group. However, the combination of PT and OT improved both clinical parameters and the reduction in MMP-9 levels. It was also shown that the degree of malocclusion also significantly affects MPP-9 levels.

d’Apuzzo F. et al. [32] studied the composition, structure, and molecular interaction of gingival crevicular fluid (GCF) and periodontal ligament (PDL) during orthodontic tooth movement (OTM) with optical vibrational techniques to put in place more personalized treatments, reducing any side effects. At the same time, Jeon H. H. and colleagues [33] examined alveolar bone remodeling during the OTM and the involved mechanisms, such as mechanosensing, sterile inflammation-mediated osteoclastogenesis, and tensile force-induced osteogenesis. They found that the cells most involved in the OTM response are periodontal fibroblasts, mesenchymal stem cells, osteoblasts, osteocytes, and osteoclasts. At the same time, intercellular signals that stimulate the OTM include RANKL, TNF-α, DKK1, sclerostin, TGF-β, and BPM.

Finally, Contaldo M. et al. [34] focused on the biological and microbiological changes related to the OT to highlight further correlations between orthodontic devices and qualitative and quantitative changes in the oral microbiota. Orthodontic patients reported significant differences in supragingival and subgingival plaque during the entire OT. Some fixed appliances, such as bonded molar brackets or elastomeric ligatures, showed high risks of periodontal disease and tooth decay for patients.

## 3. Conclusions

To summarize, these data provide the foundation for establishing the best possible diagnosis and serve as a reference point for future scientific study in orthodontics. Analyses and surveys conducted using new analysis equipment and cutting-edge technology demonstrated great repeatability and validity when compared to manual approaches, which had previously been regarded as the gold standard. In reality, the findings of this research provide a trustworthy foundation for treatment planning and can assist doctors in implementing therapy for all types of patients. Further study is needed to corroborate these assumptions, which should be chosen based on varied individual scenarios while constantly taking biological variety into account. Nonetheless, while AI is certainly useful to orthodontists and other health professionals, doctors will always be responsible for making ultimate health choices.
